# Sunflower Genetics from Ancestors to Modern Hybrids—A Review

**DOI:** 10.3390/genes9110528

**Published:** 2018-10-30

**Authors:** Aleksandra Radanović, Dragana Miladinović, Sandra Cvejić, Milan Jocković, Siniša Jocić

**Affiliations:** Institute of Field and Vegetable Crops, 21000 Novi Sad, Serbia; dragana.miladinovic@ifvcns.ns.ac.rs (D.M.); sandra.cvejic@ifvcns.ns.ac.rs (S.C.); milan.jockovic@ifvcns.ns.ac.rs (M.J.); sinisa.jocic@ifvcns.ns.ac.rs (S.J.)

**Keywords:** sunflower, *Helianthus*, domestication, breeding, genetic analysis

## Abstract

Domestication and the first steps of sunflower breeding date back more than 4000 years. As an interesting crop to humans, sunflower underwent significant changes in the past to finally find its place as one of the most significant oil crops today. Substantial progress has already been made in understanding how sunflower was domesticated. Recent advances in molecular techniques with improved experimental designs contributed to further understanding of the genetic and molecular basis underlying the architectural and phenotypic changes that occurred during domestication and improvements in sunflower breeding. Understanding the domestication process and assessing the current situation concerning available genotypic variations are essential in order for breeders to face future challenges. A review of the tools that are used for exploring the genetic and genome changes associated with sunflower domestication is given in the paper, along with a discussion of their possible implications on classical sunflower breeding techniques and goals.

## 1. Introduction

All modern domesticated sunflowers (*Helianthus annuus* L.) can be traced back to a single center of domestication in the interior mid-latitudes of eastern North America [1]. The beginnings of domestication and the first steps of sunflower breeding date back to the time when it was cultivated by native Americans over 4000 years ago [2]. Today, sunflower is the fourth most important oil crop in the world, after palm, soybean, and rapeseed, and the second most important in Europe, after rapeseed. Worldwide oil production shows a growing trend, leading to the rise of sunflower production [3].

Substantial progress has already been made in understanding how sunflower was domesticated. Recent advances in molecular techniques with improved experimental designs, including association mapping, genome-wide association studies, and candidate gene approaches, further contributed to our understanding of the genetic architecture of novel sunflower traits and the phenotypic changes in sunflower during domestication. The recently published sunflower genome sequence by Badouin et al. [4] will only add to this knowledge.

In this paper, we give a review of the genetic and genomic studies that are related to the genetic and genome changes associated with sunflower domestication, and discuss their possible implications on classical sunflower breeding techniques and goals.

## 2. Sunflower—History and Domestication

Sunflower is an annual crop. Its botanical name *Helianthus* originates from the Greek words *helios*—the sun, and *anthos*—a flower. The main reason for sunflower growing is the extraction of oil from its seeds, which makes it an important oil crop.

Archaeological findings show that the Native American started the domestication of sunflower in 4225 B.C. [5]. Sunflower was used in food (roasted kernels and flour), to obtain oil (sunscreen and hair decoration), for medical purposes (anti-inflammatory effects and diuretic), and as an ornamental plant (religious ceremonies). Since the harvest of each sunflower plant was a special operation, and any variation in the seed size was easy to see, it is logical that the plants with the largest seeds were left for planting in the following season. This was in essence a mass selection for the seed size. Burke et al. [6] found that direct selection for increased seed size played a major role in sunflower domestication. The cultivated sunflower as we know it today was most likely created by mass selection from the wild *H. annuus*, which has small seeds and a branched stem.

After its introduction into Europe in 1510 [7], the sunflower was used exclusively as an ornamental plant for more than two centuries. It became an oil crop only when it reached Russia. The history of sunflower as an oil crop can be divided into three basic periods. The first period is the use of varieties created by mass selection, the second is the use of varieties created by the method of individual selection, and the third, which is still present, is the introduction of hybrids in the production of sunflower.

The phenotypic changes that sunflower has undergone during domestication largely follow the domestication syndrome. These adaptations to human cultivation include a dramatic increase in apical dominance, an increase in seed size, the loss of natural seed dispersal and seed dormancy, and the loss of self-incompatibility [8]. Hence, cultivated and wild sunflower exhibit a number of morphological differences that trace back to the original domestication event. For example, wild sunflower is characterized by a highly branched growth form with numerous, small flowering heads, and relatively small achenes (i.e., single-seeded fruits) that are released upon maturation. Cultivated sunflower, on the other hand, is completely unbranched, producing a single large head as well as relatively large achenes that remain until harvest [9]. Sunflower has also undergone both selection and genetic drift during domestication and improvement, which has reduced its genetic diversity [10,11], with modern cultivars retaining 50–67% of the diversity that is present in wild *H. annuus* populations [12,13].

Genetic studies of sunflower domestication revealed that contrary to findings in other plant species, where it was found that the phenotypic differences caused by domestication are due to a smaller number of genes with a strong effect [14,15], in sunflower, there is a larger number of genes involved in domestication, with the majority of genes showing small or moderate phenotypic effect [6,8]. Another difference between wild and cultivated sunflowers is the copy number of long terminal repeats (LTR) retrotransposons and splicing divergence [16]. A detailed list of domestication related quantitative trait loci (QTL) mapped in different crosses between cultivated and wild sunflower and primitive and wild sunflower is given in Table A1.

## 3. Seed Characteristics and Oil Content and Composition Changes

### 3.1. Achene Size and Shattering

**Figure 1:**
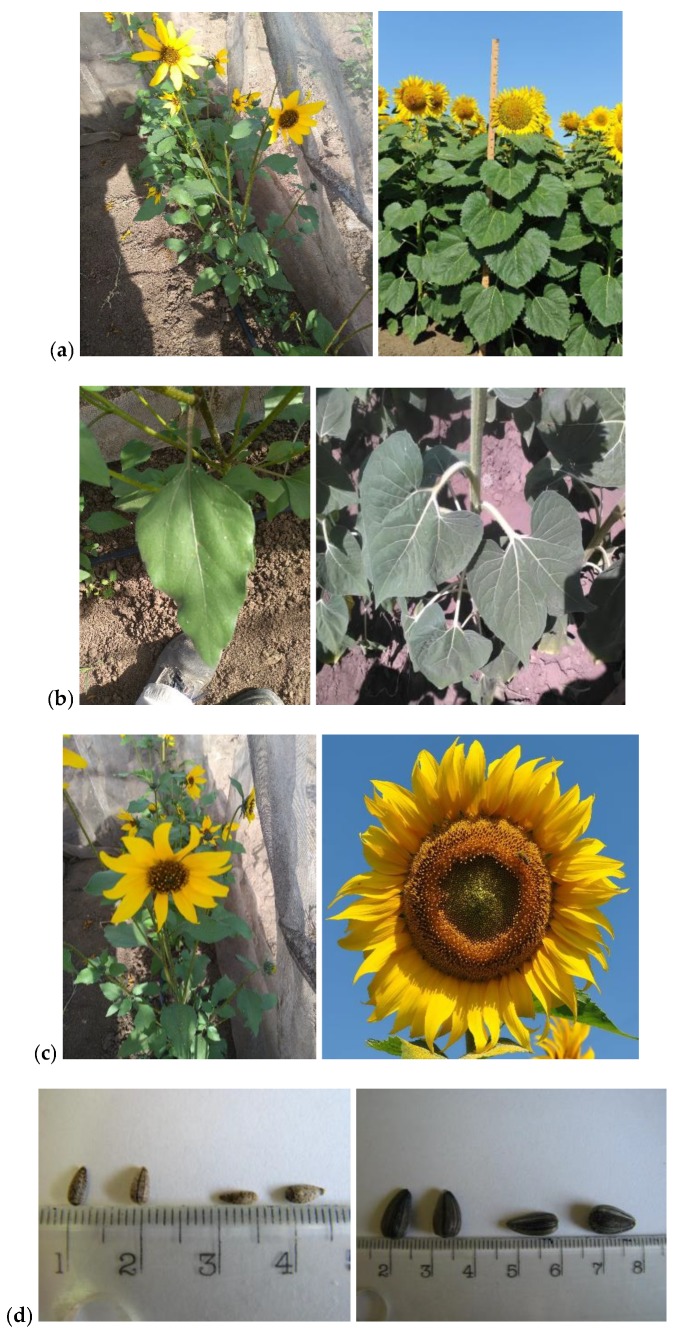
Differences in phenotypes of wild (**left**) and cultivated *Helianthus annuus* (**right**). (**a**) Plant habitus; (**b**) Leaf; (**c**) Head; (**d**) Seeds.

Wild *H. annuus* achene is of wide obovate shape, measuring 0.29 mm to 3.3 mm in width, and 0.41 mm to 6.7 mm in length [17,18]. Its color is somewhat brown, with two or three dark stripes that are variable in width [18]. However, cultivated sunflower achene is significantly larger, measuring 7 mm to 25 mm in length, and 4 mm to 13 mm in width [3] (Figure 1d). During the extensive breeding process of sunflower, hull content has decreased from 40–50% to 20–25% [19]. As outlined by Morozov [20], oil content increases by between 0.47–0.75% for every 1% of hull decrease. Achene size, weight, and shattering are some of the most important changes in sunflower, which enabled it to be used by humans. When sunflower was domesticated, its seeds increased in size and weight, while easy seed dispersal was disabled.

Selection for larger achenes was integral to sunflower domestication [6,21,22]. However, direct selection for increased seed oil in early oilseed sunflower breeding programs indirectly selected for smaller achenes, and shifted the phenotype toward the wild type [7,21,22,23,24]. Burke et al. [6] associated seven and five QTLs to achene weight and width, respectively, while only two QTLs were associated with achene length (on chromosomes 5 and 10). Chromosomes 3, 6, 9, and 10 carry QTLs for more than one achene morphology parameter, confirming the polygenic nature of these traits. Burke et al. [6] and Baack et al. [25] discovered two QTLs that have been associated with the domestication QTLs on chromosomes 6 and 10; however, the position of the QTLs was different in the two studies. Chapman et al. [26] mapped four candidate genes for selection on chromosome 10 in an interval where QTL for achene size was previously reported [6,8]. Recently, Corbi et al. [27] mapped several QTLs associated with seed mass on chromosomes 3, 4, 8 and 9. 

Concerning shattering, Burke et al. [6] found QTLs on chromosomes 11 and 17 that explained 6.6% and 5.0% of the phenotypic variation for this trait. Both QTLs expressed a dominant mode of action of the cmsHA89 allele. In contrast to this this study, Wills and Burke [8] mapped two QTLs that were associated with shattering on chromosome 4 and chromosome 10. These QTLs individually explained between 6.4–10.7% of phenotypic variation. The authors also mapped the QTLs associated with seed germination on chromosomes 12 and 15, explaining 17.3% and 17.8% phenotypic variation, respectively. Later on, Baack et al. [25] reported several QTLs associated with shattering on chromosomes 9, 13, and 16. 

### 3.2. Oil Content

Sunflower seeds are mainly used for oil extraction, which is predominantly used for human nutrition. The populations of wild *H. annuus* usually contain below 30% of oil in seed [17,28]. The first sunflower varieties with increased oil content such as Peredovik, VNIIMK 8931, Smena, and others that were created at sunflower breeding centers in the former Soviet Union had 40–45% oil content [29]. Today, most of the sunflower hybrids have 45–50% of oil in seed [29]. 

In their study, Burke et al. [9] mapped QTL controlling differences in seed oil content and composition between cultivated and wild sunflower and used the results, along with those of a previous study of domestication-related QTL, to guide a genome-wide analysis of genetic variation for evidence of past selection. They identified a QTL for oil content on LG4, and determined the mode of action of the cmsHA89 allele as partly recessive. Comparative transcriptomic analysis of the accessions of *H. annuus*, *H. petiolaris*, and *H. argophyllus*, landraces, and cultivated sunflower revealed two strongly differentiated genes involved in oil production [30]. By analyzing the sequence homology with *Arabidopsis* genes, these two putative domestication genes showed the highest homology with *AT5G49460* and *AT5G52840* genes, which encode ATP citrate lyase subunit B2, and have a function related to NADH ubiquinone oxidoreductase, respectively. The third domestication gene, the authors reported, showed homology with a gene that codes a subunit of pyruvate kinase, which is an enzyme that was involved in the conversion of carbohydrates to seed oil. In order to determine the genetic bases of seed oil content and quality, Badouin et al. [4] reconstructed a genome-scale metabolic network for the sunflower, and extracted the metabolic pathways that are involved in oil synthesis, yielding a total of 429 genes mapped onto 125 reactions, corresponding to 12 pathways.

### 3.3. Oil Composition

Sunflower oil is considered a premium quality oil. Standard sunflower oil is usually composed of polyunsaturated linoleic acid (18:2) and monosaturated oleic acid (18:1) in ratios of 70%:20%. Although the content of these two fatty acids could vary due to the effect of the environment, it is typical for sunflower oil that they jointly make about 90% of the total fatty acid content in the oil [29]. In a lower percentage, there are also unsaturated palmitic (16:0) and stearic acids (18:0), which together make up 5–15% of fatty acids. Similar oil composition has been reported for some Argentinian populations of wild *H. annuus* [17]. Additionally, in cultivated sunflower, there are also traces of monosaturated myristoleic, and mitoleic acids, as well as unsaturated myristic, arachidic, and behenic acids, and a few other fatty acids. An analysis of the changes in the fatty acid content between wild and cultivated sunflower in the progeny of cultivated sunflower cmsHA89 and wild *H. annuus* (ANN1238) showed that only palmitic fatty acid content was similar between the examined cultivated and wild sunflower [9]. The authors identified two to three QTLs that were associated with palmitic, stearic, oleic, and linoleic fatty acid content. Chromosome 6 was a common chromosome carrying a QTL for each fatty acid content in the interval between simple sequence repeat (SSR) markers ORS541 and ORS57. In their study, Premnath et al. [31] mapped the *Ol* gene to chromosome 14. They also identified two more QTLs for oleic acid content on chromosomes 8 and 9, as well as two QTLs for linoleic acid content on chromosomes 8 and 14.

Concerning oil composition, Chapman and Burke [32] discovered that seven out of the 11 genes that underlie fatty acid biosynthesis and metabolism in wild and cultivated sunflower underwent selection (*FAD2-1*, *FAD2-3*, *FAD3*, *FAD6*, *FAD7*, *FAB1*, and *FATB*). The authors selected sequences that showed orthology with *Arabidopsis* for the study, and analyzed different desaturase and thioesterase enzymes that were involved in the fatty acid conversion pathway. By examining wild, primitive, and improved genotypes, Chapman and Burke [32] were able to determine that desaturase *FAD7* was subjected to natural selection before domestication, *FAD2-3* and *FAD3* were subjected to natural selection during domestication, and *FAB1*, *FATB*, *FAD2-1*, and *FAD6* were subjected to natural selection during a period of improvement. Furthermore, a molecular analysis of “Core 12” (a group of 12 improved sunflower lines chosen from a panel of more than 400 cultivars) showed that the selective events occurred before the selection of oilseed and confectionary types was separated.

## 4. Plant Architecture Changes

### 4.1. Branching

As one of the main changes in sunflower architecture during domestication and breeding, branching has been widely investigated. This change in branching pattern could have occurred during or before the domestication process if ancient indigenous farmers had selected this trait from among wild populations [33]. Studies of the genetic basis of branching in crosses between wild and domesticated sunflower showed that it is a complex trait on which genetic background has a large effect [6]. There are many publications about the number of genes controlling branching [34,35,36,37,38].

This trait is particularly interesting in sunflower, because branching is a wild species-related trait that was lost in cultivated sunflower, only to be reintroduced from wild sunflower in restorer genotypes in order to increase the capitula number and thus ensure prolonged pollen production and successful crossing between female (cytoplasmic male sterility—*cms* line) and restorer sunflower lines.

The branching locus, B locus, was mapped on the upper part of chromosome 10 in sunflower, and in branching genotypes, this locus is in its recessive form [39]. The authors also reported several loci for domestication and post-domestication, such as oil content and achene weight, which are flanking B locus. Taking into account that branching is a complex trait in crosses between wild and cultivated sunflower and that it is a major domestication trait in sunflower, numerous studies have been conducted in order to detect the QTLs that are associated with branching. Burke et al. [6] mapped three QTLs associated with branch number on chromosomes 6, 7, and 13. Interestingly, at QTLs detected on chromosomes 6 and 13, the cmsHA89 allele had a wild-like phenotypic effect, meaning that although the respective alleles originated from the cultivated line, they produced phenotypes similar to wild-type. Mandel et al. [40] mapped 17 QTLs associated with branching on 12 chromosomes (chromosomes 2, 4, 5, 6, 7, 8, 9, 10, 12, 13, 14, and 17) by using the association mapping approach. The most important region that was associated with branching was, as expected, on chromosome 10, while other important QTLs were found on chromosome 8 and chromosome 13, and a single marker was found on chromosome 14. On chromosome 10, Mandel et al. [40] found a great variation of inserted fragments from the wild crop relative that spanned app. 25 cM in the branching haplotype, while an inserted fragment spanned app. 10 cM in the unbranched haplotype. The QTL for branch number was found on chromosome 10 between SSR markers ORS878 and HT419, in addition to the QTLs found on chromosomes 13, 16, and 17 in a cross between wild sunflower Ann1238 and domesticated primitive Hopi sunflower (USDA PI 432504) [8]. Later on, Mandel et al. [41] found that the homolog of the *LATERAL SUPPRESSOR* (*LAS*) gene exhibited positive selection during domestication, while the homolog of the *MORE AXILLARY GROWTH 2* (*MAX2*) gene exhibited it during improvement. For another homolog gene, *ISOPENTENYL TRANSFERASE 5* (*IPT5*), timing could not be determined. Out of the three gene homologs, the authors determined the position of one, *MAX2*, on chromosome 17, which co-localized with a known QTL for branching. Baute et al. [30] found a homolog of *AT3G54610* on chromosome 10 that was associated with branching. In sunflower, this gene was named *HaGNAT* (histone acetyltransferase found in *GNAT* family), and was introduced from *H. annuus* var. texanus in the RHA 274 line. 

Corbi et al. [27] mapped the QTLs for branch number on chromosomes 3, 4, 9, and 12. On chromosome 3, the wild allele increased its frequency in the recombinant inbred lines (RILs) that were obtained by crossing wild and cultivated sunflower cmsHA89 (USDA Ames 3963, PI 650572) and single wild *H. annuus* var. annuus individual (ANN1238, PI 659440) (the process of obtaining RILs is also described in Baack et al. [25]. Furthermore, in the similar region of chromosome 3, Dechaine et al. [42] mapped a QTL that is associated with branch number by use of the RILs obtained by the same way as Baack et al. [25], but tested them in two locations: North Dakota and Nebraska. 

### 4.2. Stem Properties and Height

Sunflower hybrids are typically nonbranched annual plants, from 150 cm to 180 cm in height, which are distinguished from other cultivated crops by large conspicuous inflorescence containing a large number of large achenes. Unlike cultivated sunflower, wild *H. annuus* is characterized by a plant height ranging from 63 cm to 171 cm, highly branched growth form with numerous, small flowering heads, and relatively small achenes that are released upon maturation [9,43] (Figure 1a).

Stem diameter QTLs were found on chromosomes 1, 3, 6, 7, 11, and 17 [6] in an F_3_ cross between cmsHA89 and wild *H. annuus* var. annuus individual that was collected at Keith County, Nebraska, United States (USA) (Ann1238), while Baack et al. [25] found one stem diameter QTL on chromosome 3, and Dechaine et al. [42] mapped a QTL associated with stem diameter on chromosome 13 by analyzing the RILs derived from the same parental material as in Burke et al. [6] and testing it in two different environments (North Dakota and Nebraska). HT568 and CRT504 were reported as the flanking markers to this QTL. It should be noted that Baack et al. [25] and Dechaine et al. [42] detected QTLs in two locations, one of which was the same, Nebraska (the Cedar Point Biological Station), which is also the same location where the wild parent was collected. In both studies, the researchers used RILs that were obtained from a cross cmsHA89 and wild *H. annuus* var. annuus (Ann1238). Later on, Wills and Burke [8] mapped the QTLs for stem diameter on chromosomes 1, 2, 3, 8, and 15; however, they were not in the same position as the previously mapped QTLs. Out of all of the identified QTLs, only the QTL that was mapped by Corbi et al. [27] on chromosome 13 falls in the similar region as the one mapped by Dechaine et al. [42]. The other QTLs are mapped in different positions compared to the already identified QTLs, and are located on chromosomes 1, 3, and 9.

Burke et al. [6] found QTLs that were associated with plant height on chromosomes 3, 6, 7, 10, 13, and 17, the majority of which were in the “wrong” direction (Burke et al., 2002), while Wills and Burke [8] mapped five QTLs that were associated with plant height, one of which, on chromosome 15, explained the highest percentage of phenotypic value compared to the QTLs on other chromosomes: 39.4%. Furthermore, Baack et al. [25] found five QTLs associated with plant height on chromosomes 3, 6, 7, 8, and 10; however, none of these QTLs was common for the two tested environments (North Dakota and Nebraska). Corbi et al. [26] found new QTLs for stem height on chromosomes 3, 8, and 13, some of which were in close proximity to the already reported QTLs (QTL on chromosome 3 with previously reported QTLs, and QTL on chromosome 8 with QTL reported by Baack et al. [25].

### 4.3. Leaf Properties

In sunflower, there is significant variability in all of the leaf characteristics, such as petiole angle, petiole length, total number of leaves per plant, and total leaf area per plant [44,45] (Figure 1b). There is considerable difference in the leaf number per plant in connection to the vegetation period, as it is described that early genotypes have a lower leaf number per plant, while genotypes with longer vegetation have a higher number of leaves per plant [44].

Burke et al. [6] reported two QTLs on chromosomes 12 and 13 for leaf shape that expressed dominant and additive modes of action (of the cultivated, cmsHA89, allele). The leaf size QTLs that were found on chromosomes 3–5 and 9 expressed recessive and partially recessive mode of action, while two QTLs expressed overdominance. Three of the QTLs on chromosomes 1, 9, and 17 for the number of leaves on the main stem expressed a partially dominant mode of action of the cultivated allele, while the remaining two on chromosomes 6 and 7 expressed additive and partially recessive modes of action. Two peduncle-length QTLs were found on chromosome 17, and one was found on chromosome 10. Baack et al. [25] found a QTL that was associated with leaf number and leaf moisture content on the lower end of chromosome 6 that was common in two different locations (Nebraska and Indiana) in a cross between cmsHA89 and wild *H. annuus* var. annuus. 

Dechaine et al. [42] mapped QTLs associated with leaf area while testing RILs (cmsHA89xwild *H. annuus* var. annuus (Ann1238)) in North Dakota and Nebraska, and found no mutual QTLs for the two locations. Among others, the authors mapped a QTL on chromosome 5 that was present in two locations; however, it was mapped in different positions on chromosome 5. Namely, a QTL from North Dakota was located between SSR markers ORS1120 and HT440 on the lower end of the chromosome, while a QTL discovered in North Dakota was mapped in the middle of the linkage group flanking ORS825 and ORS1220. Unlike the studies mentioned above and in which a cross between cmsHA89 and wild *H. annuus* was used, Wills and Burke [8] used a domesticated Hopi sunflower landrace to cross with wild *H. annuus*, and mapped the QTLs for a number of main stem leaves on chromosomes 6, 7, 9, and 15, and for leaf size on chromosomes 5, 8, 10, and 14–16. The QTL for the number of main stem leaves on chromosome 15 explained 57% of the phenotypic variation, and was the nearest to the SSR marker ORS687. Recently, Corbi et al. [27] mapped several new QTLs associated with leaf number on chromosomes: 4, 7, 11, 12, 14, and 16, explaining 5.87% to 12.04% of the phenotypic variation, and mapped proximal flanking markers (mainly single nucleotide polymorphism (SNP) markers, but also SSR or insertion-deletion polymorphisms (INDEL) markers) to the reported QTLs.

### 4.4. Head Properties

The domestication of sunflower significantly changed head properties. Besides being monocephalic, cultivated sunflower has a significantly larger head diameter than wild sunflower (Figure 1c). The head diameter in wild sunflower ranges from 2.4 cm to 8 cm, while the head diameter in cultivated sunflower falls between 20–30 cm [43,45,46]. Furthermore, there is great variability in the head shape of cultivated sunflower, which can be flat, concave, or convex, as determined by breeder preference and head inclination. Depending on the regions where cultivated sunflower is grown, there is a significant variability of head inclination, which is connected to sun burns, bird damage, and head rot diseases [44,45].

Head diameter-associated QTLs were reported on chromosomes 4, 5, and 13 [6]. Baack et al. [25] reported several QTLs associated with head diameter on chromosomes 4, 6, 19, and 14, of which only the position of one QTL on chromosome 14 overlapped for the two tested environmental conditions (Nebraska and Indiana), which was mapped near marker HT319. Wills and Burke [8] mapped QTLs associated with the number of heads on chromosomes 6, 8, 10, 13, 16, and 17 (two QTLs were mapped on chromosome 13), while seven QTLs were associated with disc diameter on chromosomes 1, 6, 8, 9, 10, 14, 15, and 17.

### 4.5. Floral Properties

The domestication of sunflower favored increased floral size, such as an increase in the number of ray flower and ray flower length. Five and three QTLs for the number of ray flowers and ray size were mapped in sunflower by Burke et al. [6]. The majority of the QTLs for ray size were in the “wrong” direction, while chromosomes 6 and 9 harbored QTLs for both traits. Wills and Burke [7] detected six QTLs for ray flower number; however, none of the QTLs were common between this and a previous study conducted by Burke et al. [6].

## 5. Changes in Reproductive Strategy

The domestication of sunflower was marked by a loss of self-incompatibility [22,23], favoring the pollination of one sunflower plant with the pollen of another and decreased seed dormancy [24]. These traits have been lost or partly lost during domestication and breeding; thus, cultivated sunflower is self-compatible, and has a short-lived seed dormancy [47,48].

Ghandi et al. [49] were the first to examine the QTLs for self-incompatibility and self-pollination in sunflower. The authors used a BC_1_ family obtained from a cross between an inbred line NMS373 (self-pollinated, non-dormant) and wild sunflower ANN1811 (self-incompatible, dormant). The authors mapped S locus (self-incompatibility locus) as an incomplete dominant allele on the lower end of chromosome 17. The authors also argued that one of the QTLs detected for the number of selfed seeds by Burke et al. [7] on chromosome 17 was, in fact, this S allele. The SSR marker that is tightly linked to this locus was ORS735. Additionally, Ghandi et al. [49] mapped three QTLs associated with self-pollination on chromosomes 6, 15, and 17, and three QTLs associated with seed dormancy on chromosomes 3, 11, and 15. Wills and Burke [8] also mapped one QTL for number of selfed seeds, which is directly correlated with self-pollination, on chromosome 17, as well as on chromosomes 1, 8 and 12.

Burke et al. [6] found two major QTLs associated with the number of selfed seeds on the lower half of chromosome 17. These QTLs explained 12.7% and 68% of the phenotypic variation, and both were found to be partially recessive. In addition to the QTL associated with number of selfed seeds on chromosome 17, Wills and Burke [8] mapped three more QTLs associated with this trait on chromosomes 1, 8, and 12, in a different cross between wild and primitive sunflower.

## 6. Life Cycle Shift

Flowering time is one of the most important domestication traits, especially bearing in mind that it influences the success of the crop [4,50,51]. Wild sunflower is highly diverse when it comes to flowering, and it has a variable flowering time [22]. Selection favored consistent flowering time; however, a late flowering date was favored in the early stages of domestication in primitive sunflower [22], while modern cultivated sunflower is characterized by relatively early flowering, and it is abundant in photoperiod response [52,53]. Consequently, flowering time is one of the most investigated domestication traits in sunflower.

Burke et al. [6] reported 10 QTLs associated with days to flowering, five of which expressed an additive mode of action (QTLs on chromosomes 1, 6, 8, and two QTLs on chromosome 9). Three QTLs found on chromosome 8 and chromosome 17 showed a dominant mode of action, while the QTLs on chromosomes 4 and 7 expressed underdominance and partial recessiveness. Lai et al. [54] mapped a locus HT160 on chromosome 8. Based on homology, this locus was predicted to be the APETALA2-like protein, and was previously reported as a QTL associated with flowering time and achene size [6,55]. 

Wills and Burke [8] mapped QTLs for flowering time on three chromosomes—6, 7, and 15—in a cross between wild sunflower Ann1238 and domesticated Hopi sunflower (USDA PI 432504). The QTL on chromosome 15 was not identified in any other studies, and it explained 46.9% of the phenotypic variation. The authors reported that SSR marker ORS687 was the closest marker to this QTL. Later on, Chapman et al. [26] mapped five candidate genes on chromosome 7 in the interval where the QTLs for flowering time and the number of main stem leaves were mapped previously [6,8]. Two out of the five candidate genes, c1921 and c2588, that were mapped by Chapman et al. [26] showed homology with the genes that code a DNA-binding with one finger (Dof)-like protein and a protein with the *INDETERMINATE* domain, respectively, both of which have been shown to be involved in flowering in other plant species [56,57]. Baack et al. [25] reported QTLs for flowering date on the lower end of chromosomes 6 and 9 and the upper part of chromosome 14, which were common for two different environmental conditions (Nebraska and Indiana) in a cross between cmsHA89 and wild *H. annuus* var. annuus. The QTL on chromosome 9 was a common QTL for flowering date in studies reported by Burke et al. [6] and Baack et al. [25].

Dechaine et al. [42] enriched a previously reported map by adding the domestication and/or improvement loci identified by Chapman et al. [26] to the SSR markers that were used by Baack et al. [25], and found QTLs associated with flowering time on chromosomes 1, 6, 7, 8, 14, and 17 that described between 6.56–22.67% of the phenotypic variation.

Blackman et al. [50,58] conducted a comprehensive study of the different genes that have undergone changes during domestication and improvement. The authors used an integrated candidate approach by analyzing the homology with genes of known function and the positions of QTLs associated with flowering times that have already been reported in the literature. In addition, the authors determined that the expression of duplicated homologs of the *FLOWERING LOCUS T* (FT) in sunflower have a role in sunflower domestication. Four FT-like paralogs have been isolated (*HaFT1-4*) in the sunflower genome. *HaFT1* was under selection in domestication, while the other paralogs were selected during improvement. *HaFT1-3* was mapped on chromosome 6 and *HaFT1* underlies a major flowering time QTL. The *TERMINAL FLOWER 1* paralog in sunflower, *HaTFL1*, was also under selection during improvement, and was mapped on chromosome 7. The authors analyzed the expression and interactions between flowering time and associated genes [50,58].

With the availability of new technologies, Mandel et al. [40] mapped the QTLs associated with days to flower by use of the Illumina Infinium 10 k SNP array for sunflower, and found significant associations for this trait in 10 genomic regions located on 8 of 17 sunflower chromosomes. Significant associations were found on chromosomes 1, 3, 4, 9, 10, 12, 13, and 17, some of which were novel QTLs (on chromosomes 1, 3, 4, 10, 12, 13). A year later, Mandel et al. [41] used a candidate gene approach and analyzed several genes related to flowering type to detect the ones that underwent changes during selection. One of these was *PHYTOCHROME B*, which was marginally significant during improvement according to previous study [50]. Later on, Baute et al. [30] found regions on chromosome 1 and chromosome 10 that could be linked to flowering time in sunflower, which were homologs of *ATMYB59* and *AT5G62430* (a cycling DOF factor), respectively. Recently, Corbi et al. [27] found QTLs associated with flowering time on chromosomes 1, 6, 7, 14, and 17, explaining 7.48–27.37% of the phenotypic variation and mapped closest markers (SNPs, SSR, and INDEL) to the QTLs. The QTL on chromosome 6 was flanking previously mapped genes *HaFT1* and *HaFT2* [50].

## 7. Other Traits of Cultivated Sunflower

Sunflower has become one of the most important oil crops in the world. So far, significant results have been achieved in sunflower breeding: the model of the hybrid has been created and breeding directions have been established; the genetic pool of the cultivated sunflower has been created and a rich collection of wild species of the genus *Helianthus* has been created; methods of biotechnology have been developed, and hybrids for different uses have been created. 

Dimitrijević and Horn [59] gave a detailed review of the basic directions of sunflower breeding and the future perspectives of using modern molecular tools to detect and exploit genetic diversity and facilitate sunflower hybrid breeding. With current forecasts of population increase and climate change, it is assumed that current sunflower production is insufficient for future needs. In order to overcome this, the future directions of sunflower breeding will be focused on complex traits: (1) yield; (2) quality characteristics of seed; and (3) resistance to biotic constraints.

Seed yield remains the most important objective of sunflower breeding. Selection for higher seed yield and other traits should begin during inbred line creation by defining the effects of heterosis and analyzing and evaluating the correlations among them to develop a productive hybrid with the desired traits [60]. Therefore, the selection of high-yielding parental lines is an important prerequisite. Both general combining ability (GCA) and special combining ability (SCA) may be important in parental and hybrid identification. Breeding for yield has changed from maximum possible yield under intensive agriculture to yield with resistance to abiotic stresses, moderate droughts, and shallow soil in particular, which was helped by collaboration with agronomists to produce crop models [61]. The sunflower crop has been proposed as a potential crop model for adaptation to a changing environment. In this regard, special attention should be paid to achieving yield that is as high and stable as possible under unfavorable conditions of cultivation and environment. In the future, sunflower breeding will be oriented not only to increased yield, but also to its adaptability in the form of shortening the vegetation period in order to adapt to the new growing areas. In northern parts of Europe, where sunflower is not grown now, new possibilities for producing early hybrids can be expected, which would allow diversity in the existing small grains-based crop rotation system [62]. With intensified production, we can expect sunflower to be grown as a second crop, which again favors the selection of hybrids with a short growing period. In future cultivation systems, sunflower will find its place as a low-input crop that produces high yields, as it belongs to C3 plants, where increased atmospheric CO_2_ affects the growth and yield of plants mainly through increased photosynthesis and assimilation of carbon

Considering that the phenotype represents the realization of a genotype in certain environmental conditions, the increased genetic variation of plant architecture is of great importance in order to maximize the productivity within the conditions in which the plant is grown. One of the main components of seed yield is plant number per hectare, which largely depends on plant architecture. By using shorter sunflower hybrids with increased leaf area and leaf arrays that grow vertically and horizontally, plant number per hectare can be increased, and consequently, so can seed and oil yield. Also, developing hybrids with shorter petiole length, as can be seen in *H. maximiliani*, can increase the number of plants per hectare. Likewise, an interesting approach can be increasing the number of capitate glandular trichomes, which are deficient in cultivated sunflower compared to wild, as they are considered to be effective defense components that act against some herbivorous insects such as sunflower moth and larvae [63]. Wild *Helianthus* species represent a diversified source of agronomically important traits that can contribute to future modeling of the sunflower plant in order to improve plant architecture and maximize productivity [64,65].

Quality has become a challenging target for sunflower breeders worldwide. Modern sunflower breeding requires great attention to altering oil quality. Although sunflower oil is one of the finest plant oils, sunflower breeders have reacted to market demands and managed to make significant changes in the quality of sunflower oil, in terms of fatty acid composition and tocopherol content [66]. Sunflower genotypes containing high, mid, and low levels of saturated fatty acids, mid and high oleic acid content, as well as containing beta, gamma, and delta-tocopherols, enabled the creation of more oil profiles with different fatty acids and tocopherol combination than in any other oil crop. New traits of fatty acid and tocopherol content show significantly higher stability compared to environmental factors, and they are controlled by a small number of genes. Consequently, they can easily be used in breeding programs with the purpose of developing hybrids with different oil quality. New genes combined with the existing genes for oil quality enable the accumulation of several different traits in one genotype, which allows the development of hybrids with different oil qualities, which are used for various purposes. The combination of several quality traits in a single phenotype will enable tailoring specialty oils, providing essentially “new oilseed crops” for specific uses in food and non-food industries, thus guaranteeing a promising future for sunflower on the global world market [67]. Sunflower meal is rich in proteins and good row material for feed. Breeding for the increased nutritive quality of sunflower meal aims to increase the protein (which can be over 20% in current hybrids) and lysine content, which is deficient in sunflower, as well as reduce the fiber content to improve meal digestibility [68].

Breeding for resistance to diseases is another significant aspect. Diseases have always been a limiting factor of sunflower production. Increased resistance to dominant diseases is one of the basic tasks of sunflower breeders, which must be solved in order to make the sunflower ready for the upcoming climate change. That is why it is necessary to achieve long-term tolerance or resistance to a specific pathogen [69]. Although the number of pathogens that are known to attack sunflower is relatively high, only a handful to a dozen are considered important, depending on the region and cultivar [70]. An exciting and challenging area of sunflower breeding research would be to develop hybrids with built-in genetic resistance to *Plasmopara halstedii*, *Diaporthe*/*Phomopsis helianthi*, *Puccinia helianthi*, *Sclerotinia sclerotiorum*, *Verticillium dahliae*, *Phoma macdonaldi*, *Macrophomina phaseoli*, *Botrytis cinerea*, *Albugo tragopogis*, *Rhizopus* spp., *Alternaria* spp., *Erysiphe cichoracearum*, *Septoria helianthi*, and *Fusarium* spp., and resistance to the parasitic weed broomrape (*Orobanche cumana*). A major obstacle and breeding for disease resistance is the constant emergence of new races of pathogens. Wild sunflower species have remained as genetic sources of resistance, and resistance genes have been successfully transferred to cultivated sunflower. Breeding programs aiming to produce genotypes with strong and durable disease resistance should combine different resistance genes that have minimal adverse effects on other desired traits. The presence of multiple resistance genes may offer greater evolutionary impedance than a single resistance gene, since a pathogen would have to develop mutations in all of the effectors that are recognized by the resistance genome complement in order to overcome complex resistance [71].

## 8. Future Prospects and Implications for Breeding

The QTLs reported in this review are associated with major domestication traits. These QTLs can be used as diagnostic markers in tracking introgression from wild into cultivated sunflower, and eliminating unwanted sequences surrounding the gene of interest during introgression. Also, another application would be to identify the types of crop-like traits that are favored in the wild if they are subjected to manipulation [27]. Linkage maps obtained from wild and cultivated sunflower crosses can differ from a cultivated sunflower cross due to suppressed recombination, as reported by Wills and Burke [8], making it difficult to compare QTLs obtained in different crosses. As QTLs are also highly environmentally dependent, all of these QTLs should be further validated in different crosses and by association analysis.

The real breakthrough in sunflower molecular biology was achieved with the publishing of the sunflower genome sequence [4]. Further insight into the domestication process would be achieved by sequencing the wild *H. annuus* genome, preferably through choosing from the population used in the majority of QTL analysis of sunflower domestication, such as Ann1238. This sequence could be further used to define the location of QTLs associated in domestication that have been previously reported (and mentioned in this review), in order to gain more insight into the important metabolic pathways, as was done with the cultivated sunflower, and enable the replacement of SSR markers with more precise SNPs.

## Appendix A

**Table A1:** Positions of domestication-related quantitative trait loci (QTL) mapped in different crosses between cultivated and wild sunflower and primitive and wild sunflower.

Trait	Chr	Nearest Marker or Flanking Markers	Position (cM)	LOD Interval ^a^	PVE ^b^	Reference
**Days to flower**	1	ORS605	17.2	10.8–21.5	3.0	Burke et al. (2002)
	1	HT1018, c1774	-	0.0–21.0	10.8	Dechaine et al. (2009)
	1	SFW09467	-	0.0–22.8	7.5	Corbi et al. (2018)
	4	ORS615C	61.3	53.9–64.4	6.5	Burke et al. (2002)
	6	ORS131	52.2	48.2–54.6	28.0	Burke et al. (2002)
	6	ORS483	57.6	53.6–57.7	7.6	Wills and Burke (2007)
	6	HT913	70.7	70.0–70.7	36	Baack et al. (2008)
	6	HT918	64.5	58.9–68.5	26	Baack et al. (2008)
	6	HT913, c2603	-	69.7–71.7	22.7	Dechaine et al. (2009)
	6	HT913	-	96.7–100.1	27.4	Corbi et al. (2018)
	7	ORS331	2.0	0.0–21.7	9.5	Burke et al. (2002)
	7	ORS1041	1.0	0.0–5.3	2.5	Wills and Burke (2007)
	7	ZVG29, c1533	-	0.0–9.0	10.3	Dechaine et al. (2009)
	7	ZVG29	-	0.0–9.0	17.3	Corbi et al. (2018)
	8	ORS70	52.7	49.3–59.0	5.5	Burke et al. (2002)
	8	ORS536A, ORS154A	79.3	77.0–81.3	6.4	Burke et al. (2002)
	8	HT71, ORS70	-	26.8–50.7	6.6	Dechaine et al. (2009)
	8	SFW01442	-	44.6–78.9	13.9	Corbi et al. (2018)
	9	ORS428, ORS64	11.3	0.0–18.8	5.6	Burke et al. (2002)
	9	ORS176	53.5	49.5–54.3	3.3	Burke et al. (2002)
	9a	HT978	51.9	48.4–51.9	11	Baack et al. (2008)
	9b	HT978	52	38.9–52	15	Baack et al. (2008)
	14a	ORS398	12.9	9.0–19.6	10	Baack et al. (2008)
	14b	ORS398	14.8	12.0–17.6	30	Baack et al. (2008)
	14	c1666, G13K16	-	9.8–37.4	8.2	Dechaine et al. (2009)
	14	SFW02805	-	21.3–30.3	8.7	Corbi et al. (2018)
	15	ORS687	57.1	57.0–58.2	46.9	Wills and Burke (2007)
	17	ORS727	39.2	35.6–42.6	19.9	Burke et al. (2002)
	17	ORS204A	58.9	50.9–64.9	21.8	Burke et al. (2002)
	17	ORS735	37.5	33.3–39.5	9.0	Baack et al. (2008)
	17	ORS561, ORS735	-	35.5–43.3	7.2	Dechaine et al. (2009)
	17	SFW02587	-	73.2–78.4	16.0	Corbi et al. (2018)
**Stem diameter**	1	ORS718A, ORS53	14.8	4.8–39.5	7.8	Burke et al. (2002)
	1	HT1018	7.0	4.6–10.4	10.0	Wills and Burke (2007)
	1	ORS371, CRT391	-	23.0–39.1	12.7	Dechaine et al. (2009)
	1	ORS371	-	49.7–55.0	8.9	Corbi et al. (2018)
	2	ORS925	1.7	0.0–15.0	3.0	Wills and Burke (2007)
	3	ORS333B	31.9	20.7–39.9	13.4	Burke et al. (2002)
	3	ORS665	3.4	0.0–9.9	6.5	Wills and Burke (2007)
	3	HT441	29.0	18.5–37.5	15	Baack et al. (2008)
	3	HT1031, ORS949	-	4.0–36.4	7.5	Dechaine et al. (2009)
	3	SFW01698	-	47.9–60.8	7.7	Corbi et al. (2018)
	4	ORS963, HT298	-	0.0–10.0	6.5	Dechaine et al. (2009)
	6	ORS131	52.2	46.4–60.6	4.4	Burke et al. (2002)
	7	ORS331, ORS143	0.0	0.0–5.5	3.9	Burke et al. (2002)
	8	HT668	43.8	37.8–46.8	8.0	Wills and Burke (2007)
	9	SFW04878	-	116.1–118.1	7.1	Corbi et al. (2018)
	10	ORS878, HT347	-	0.0–24.2	7.1	Dechaine et al. (2009)
	11	ORS210	41.5	35.1–47.0	4.5	Burke et al. (2002)
	12	c0019, c3115	-	36.0–49.9	8.9	Dechaine et al. (2009)
	13a	HT568, CRT504	-	0.0–24.8	10.6	Dechaine et al. (2009)
	13b	HT568, CRT504	-	0.0–28.8	9.9	Dechaine et al. (2009)
	13	ORS511, ORS578	-	36.5–50.5	9.8	Dechaine et al. (2009)
	13	SFW05467	-	5.9–16.4	10.9	Corbi et al. (2018)
	15	ORS1141	56.4	52.4–58.2	15.7	Wills and Burke (2007)
	17	ORS204A	64.9	54.9–67.8	7.6	Burke et al. (2002)
**Height**	1	ORS716	8.0	4.6–10.0	11.9	Wills and Burke (2007)
	3	ORS134	19.3	13.3–26.7	11.3	Burke et al. (2002)
	3	HT292	8.9	4.9–15.0	13.0	Baack et al. (2008)
	3	c1144, HT1031	-	0.0–17.0	7.9	Dechaine et al. (2009)
	3	SFW07426	-	16.5–17.5	10.6	Corbi et al. (2018)
	6	ORS131, ORS608A	58.6	52.6–64.6	22.5	Burke et al. (2002)
	6	ORS483	57.6	47.6–57.7	6.4	Wills and Burke (2007)
	6	ORS57	68.5	61.7–70.5	14.0	Baack et al. (2008)
	7	ORS555, ORS671F	15.6	7.6–21.7	15.6	Burke et al. (2002)
	7	ORS331	8.4	3.9–14.3	9.0	Baack et al. (2008)
	8	ZVG37	50.7	48.5–50.7	13.0	Baack et al. (2008)
	8	HT656	-	33.3–41.6	12.5	Corbi et al. (2018)
	9	ORS1265	10.0	2.0–19.0	3.0	Wills and Burke (2007)
	10	ORS613, ORS818	50.7	46.7–62.2	5.3	Burke et al. (2002)
	10	HT347	10.2	2.8–14.4	8.0	Baack et al. (2008)
	10	HT419	30.7	26.7–32.7	9.0	Baack et al. (2008)
	13	ORS45, ORS799	35.5	29.6–36.8	5.1	Burke et al. (2002)
	13	HT568, CRT504	-	0.0–20.8	7.1	Dechaine et al. (2009)
	13	SFW05467	-	15.4–16.4	8.3	Corbi et al. (2018)
	14	HT319	16.1	10.1–18.0	3.0	Wills and Burke (2007)
	14	c2693, c5666	-	0.0–12.2	10.5	Dechaine et al. (2009)
	15	ORS687	57.1	57.0–58.2	39.4	Wills and Burke (2007)
	17	ORS717	37.6	33.6–42.6	9.2	Burke et al. (2002)
**Number of main stem leaves**	1	ORS718A, ORS474	10.8	3.3–19.2	4.6	Burke et al. (2002)
	6	ORS57, ORS131	50.2	18.2–52.2	28.1	Burke et al. (2002)
	6	ORS483	57.6	55.6–57.7	4.9	Wills and Burke (2007)
	7	ORS331, ORS555	4.0	0.0–7.5	13.3	Burke et al. (2002)
	7	ORS1041	1.0	0.0–7.3	2.7	Wills and Burke (2007)
	9	ORS176	53.5	49.5–54.3	5.2	Burke et al. (2002)
	9	HT294	19.0	13.0–39.8	5.3	Wills and Burke (2007)
	15	ORS687	57.1	57.1–58.2	57.0	Wills and Burke (2007)
	17	ORS727	39.2	35.6–42.6	9.8	Burke et al. (2002)
**Leaf number**	4	ORS674, HT221	-	73.6–84.5	13.2	Dechaine et al. (2009)
	4	SFW00857	-	105.0–121.4	11.8	Corbi et al. (2018)
	6a	HT913	70.7	69.2–70.7	25	Baack et al. (2008)
	6b	HT913	70.5	64.1–70.5	14	Baack et al. (2008)
	7	ORS331	12.4	8.4–16.5	16	Baack et al. (2008)
	7	ORS331, c1921	-	0.0–23.2	6.1	Dechaine et al. (2009)
	7	SFW01658	-	33.6–45.9	7.7	Corbi et al. (2018)
	11	SFW05043	-	113.7–118.7	6.0	Corbi et al. (2018)
	12	c0019, c3115	-	36.0–49.9	12.3	Dechaine et al. (2009)
	12	SFW09009	-	54.0–60.6	11.1	Corbi et al. (2018)
	14	SFW03980	-	58.8–77.9	5.9	Corbi et al. (2018)
	16	HT208, ORS172	-	100.2–110.3	11.4	Dechaine et al. (2009)
	16	SFW04562	-	113.6–144.2	12.0	Corbi et al. (2018)
**Leaf shape**	4	c1258, HT989	-	22.1–34.1	9.87	Dechaine et al. (2009)
	4	HT339, ORS674	-	44.1–61.6	7.70	Dechaine et al. (2009)
	6	HT769	46.0	39.1–58.0	18.0	Baack et al. (2008)
	12	ORS167, ORS256D	57.4	45.6–67.3	10.2	Burke et al. (2002)
	12	c3115, HT490	-	40.0–60.1	9.9	Dechaine et al. (2009)
	13	ORS388	4.0	0.0–10.0	21.0	Burke et al. (2002)
	16	ORS899	20.0	8.4–31.9	19.0	Baack et al. (2008)
**Leaf size (area)**	3	ORS488, ORS244B	67.2	55.3–77.2	11.9	Burke et al. (2002)
	3	HT441	29.0	19.7–41.0	11.0	Baack et al. (2008)
	3	HT1031, ORS949	-	5.3–42.4	6.6	Dechaine et al. (2009)
	4	ORS146	34.6	29.1–38.6	5.0	Burke et al. (2002)
	4	ORS963, HT298	-	0.0–10.0	6.1	Dechaine et al. (2009)
	5	ORS315B, ORS547	10.0	0.0–20.6	8.3	Burke et al. (2002)
	5	ORS852	31.6	21.6–44.5	9.1	Wills and Burke (2007)
	5	ORS852, ORS1120	-	19.1–51.1	8.6	Dechaine et al. (2009)
	5	ORS1120, HT440	-	43.1–67.0	7.1	Dechaine et al. (2009)
	8	ORS1161	35.6	32.4–35.8	5.6	Wills and Burke (2007)
	9	ORS176	51.5	43.5–54.3	7.9	Burke et al. (2002)
	10	ORS427	15.8	7.8–18.9	4.4	Wills and Burke (2007)
	10	c1700, ORS878	-	2.0–20.2	10.0	Dechaine et al. (2009)
	13	HT568, CRT505	-	0.0–29.7	9.1	Dechaine et al. (2009)
	14	ORS307	10.1	3.1–18.0	4.9	Wills and Burke (2007)
	15	ORS1141	57.0	50.5–58.2	3.7	Wills and Burke (2007)
	16	ORS407	45.4	37.4–60.1	5.1	Wills and Burke (2007)
**Leaf ratio**	7	ZVG29	-	0.0–8.0	12.4	Corbi et al. (2018)
	12	SFW09009	-	57.0–60.7	14.4	Corbi et al. (2018)
**Peduncle length**	10	ORS613	50.7	44.7–60.2	7.0	Burke et al. (2002)
	17	ORS727	39.2	35.6–41.2	4.7	Burke et al. (2002)
	17	EG825	48.9	44.6–65.8	5.7	Burke et al. (2002)
**Branch number**	3	c1144, HT1031	-	5.3–23.0	13.7	Dechaine et al. (2009)
	3	HT1031, ORS949	-	4.0–42.4	7.9	Dechaine et al. (2009)
	3	M23M12	-	31.3–45.0	12.2	Corbi et al. (2018)
	4	SFW01149	-	55.9–63.0	8.9	Corbi et al. (2018)
	6	ORS57	36.4	26.4–46.4	11.3	Burke et al. (2002)
	7	ORS143	5.5	0.0–7.3	8.8	Burke et al. (2002)
	9	CYC5B, ORS176		25.5–55.6	8.3	Dechaine et al. (2009)
	9	CYC5A		94.2–105.2	9.9	Corbi et al. (2018)
	10	ORS437	7.1	9.8–24.8	4.6	Wills and Burke (2007)
	12	c3115, HT490	-	32.0–58.1	11.2	Dechaine et al. (2009)
	12	SFW00213	-	59.7–80.8	17.7	Corbi et al. (2018)
	13	ORS995, ORS45	27.6	19.6–35.5	7.0	Burke et al. (2002)
	13	HT848	0.0	0.0–17.6	5.2	Wills and Burke (2007)
	16	ORS899	30.1	22.0–36.1	7.0	Wills and Burke (2007)
	16	HT208, ZVG75b	-	86.2–110.3	10.1	Dechaine et al. (2009)
	17	ORS737	22.0	16.0–30.1	8.4	Wills and Burke (2007)
**Number of heads**	3	HT1031, ORS949	-	11.31–45.31	7.0	Dechaine et al. (2009)
	6	ORS1229	41.1	22.8–53.6	3.1	Wills and Burke (2007)
	6	HT913	70.5	69.8–70.5	9.0	Baack et al. (2008)
	7	ORS671F	17.6	7.5–21.7	9.4	Burke et al. (2002)
	8	ORS147	29.5	19.5–35.2	5.9	Wills and Burke (2007)
	8	HT656	-	32.3–48.6	9.4	Corbi et al. (2018)
	9	ORS176	53.5	49.5–54.3	10.4	Burke et al. (2002)
	9	HT978	52.0	47.0–52.0	9.0	Baack et al. (2008)
	10	ORS437	15.8	11.8–18.9	28.1	Wills and Burke (2007)
	12	ORS167	55.4	37.6–63.4	8.0	Burke et al. (2002)
	12	c5456, c0019	-	30.5–61.1	10.8	Dechaine et al. (2009)
	12	SFW03117	-	53.0–77.4	12.8	Corbi et al. (2018)
	13	ORS995, ORS45	25.6	19.6–33.5	11.4	Burke et al. (2002)
	13	HT848	0	0.0.2.0	5.3	Wills and Burke (2007)
	13	ORS317	15.6	5.6–27.6	6.5	Wills and Burke (2007)
	16	ORS993	34.1	28.1–45.4	3.4	Wills and Burke (2007)
	17	ORS811	44.6	39.2–62.9	8.6	Burke et al. (2002)
	17	ORS735	24.8	14.0–32.8	3.4	Wills and Burke (2007)
**Number head/branch**	6	ORS374, ORS57	54.6	46.4–64.6	6.5	Burke et al. (2002)
	9	ORS176	53.5	43.5–54.3	4.9	Burke et al. (2002)
	12	ORS167	53.4	35.6–61.4	7.3	Burke et al. (2002)
	16	ORS343, ORS258A	60.1	38.6–68.9	7.6	Burke et al. (2002)
	17	EG825	46.6	42.6–58.9	8.4	Burke et al. (2002)
**Disc diameter**	1	HT39	14.4	0.0–18.3	4.4	Wills and Burke (2007)
	4	ORS785	51.9	45.3–57.9	4.6	Burke et al. (2002)
	4	ORS366	16.3	10.1–23.0	11.0	Baack et al. (2008)
	5	ORS315B, ORS240	12.0	0.0–21.5	5.7	Burke et al. (2002)
	6	ORS381	53.6	45.6–57.7	4.9	Wills and Burke (2007)
	6	HT913	70.4	69.4–70.4	11.0	Baack et al. (2008)
	8	ORS456	35.2	32.4–45.8	9.0	Wills and Burke (2007)
	9	ORS1265	17.0	8.0–22.5	7.7	Wills and Burke (2007)
	9	HT978	52.0	48.4–52.0	16.0	Baack et al. (2008)
	10	ORS437	13.8	7.8–17.1	13.0	Wills and Burke (2007)
	13	ORS388	0.0	0.0–8.0	6.0	Burke et al. (2002)
	14	ORS307	12.1	0.0–18.0	5.6	Wills and Burke (2007)
	14a	HT319	20.6	14.4–29.9	13.0	Baack et al. (2008)
	14b	HT319	20.6	11.5–30.7	9.0	Baack et al. (2008)
	14	c0211, HT528	-	13.1–47.4	10.2	Dechaine et al. (2009)
	15	ORS7	50.5	35.1–57.1	4.3	Wills and Burke (2007)
	17	ORS565	4.0	0.0–10.0	5.5	Wills and Burke (2007)
**Number of ray flowers**	1	ORS662, ORS53	35.5	25.5–41.5	7.8	Burke et al. (2002)
	1	HT446	12.0	6.0–22.9	12.0	Baack et al. (2008)
	4	HT664	12.3	6.2–18.1	13.0	Baack et al. (2008)
	5	ORS505	19.6	8.5–29.6	6.7	Wills and Burke (2007)
	6	ORS57	48.2	40.4–64.6	7.2	Burke et al. (2002)
	7	ORS143	4.0	0.0–9.6	10.1	Burke et al. (2002)
	8	ORS147	32.4	23.5–41.8	3.1	Wills and Burke (2007)
	9	ORS176	53.5	43.5–54.3	5.9	Burke et al. (2002)
	10	ORS534	13.8	4.0–22.9	3.6	Wills and Burke (2007)
	12	HT466	72.3	65.7–72.8	4.3	Wills and Burke (2007)
	13	ORS388	0.0	0.0–8.0	6.9	Burke et al. (2002)
	14	HT319	12.8	7.8–19.0	12.0	Baack et al. (2008)
	15	ORS687	57.1	48.5–58.2	13.1	Wills and Burke (2007)
	16	HT52	74.8	54.5–83.9	9.0	Baack et al. (2008)
	17	ORS735	26.8	10.0–33.7	4.6	Wills and Burke (2007)
**Ray size**	5	ORS852, ORS1120	45.3	37.7–52.3	9.0	Burke et al. (2002)
	6	ORS374	28.4	8.3–40.4	7.7	Burke et al. (2002)
	9	ORS176	51.5	43.5–54.3	8.7	Burke et al. (2002)
**Ray length**	6	HT913	70.7	69.9–70.7	28.0	Baack et al. (2008)
	9	ORS176	52.0	43.5–52.0	14.0	Baack et al. (2008)
	9	CYC5B, ORS176	-	23.5–55.6	9.1	Dechaine et al. (2009)
	13	HT568	0.0	0.0–6.0	10.0	Baack et al. (2008)
	14	HT319	14.8	10.8–20.4	13.0	Baack et al. (2008)
	16a	HT52	74.8	63.8–84.4	8.0	Baack et al. (2008)
	16b	HT52	70.8	60.1–86.0	11.0	Baack et al. (2008)
**Number of selfed seeds**	1	HT39	14.4	8.0–30.3	6.6	Wills and Burke (2007)
	8	ZVG34	15.5	6.2–29.5	5.4	Wills and Burke (2007)
	12	HT466	72.3	65.7–72.8	6.8	Wills and Burke (2007)
	17	ORS811	42.6	41.2–46.6	42.7	Burke et al. (2002)
	17	ORS204A	58.9	54.9–62.9	68.0	Burke et al. (2002)
	17	ORS735	18.0	10.0–33.7	7.2	Wills and Burke (2007)
**Achene weight**	1	HT1018	6.6	2.0–18.3	8.6	Wills and Burke (2007)
	2	ORS423, ORS279	4.8	0.0–18.0	5.9	Burke et al. (2002)
	3	ORS488	65.2	51.3–75.2	15.0	Burke et al. (2002)
	3	c1144, ORS949	-	5.3–40.4	8.9	Dechaine et al. (2009)
	3	M23M12	-	33.3–45.0	8.6	Corbi et al. (2018)
	4	SFW03768	-	0.0–8.0	10.3	Corbi et al. (2018)
	6	ORS131	52.6	48.2–64.6	5.6	Burke et al. (2002)
	6	HT913	70.7	69.8–70.7	28.0	Baack et al. (2008)
	8	ORS456	35.2	19.5–35.8	7.6	Wills and Burke (2007)
	8	HT656	-	34.0–46.6	8.7	Corbi et al. (2018)
	9	ORS176	53.5	47.5–54.3	13.7	Burke et al. (2002)
	9	CYC5B, ORS176	-	49.6–55.6	9.3	Dechaine et al. (2009)
	9	SFW04878	-	116.1–118.8	6.9	Corbi et al. (2018)
	9	HT294	19.0	6.0–35.8	4.2	Wills and Burke (2007)
	10	ORS815	39.3	28.8–44.7	12.4	Burke et al. (2002)
	10	ORS437	15.8	9.8–18.9	19.0	Wills and Burke (2007)
	10	ORS565	20.2	14.2–26.1	12.0	Baack et al. (2008)
	12	ORS502	2.0	0.0–14.0	5.7	Burke et al. (2002)
	17	ORS811	42.6	39.2–46.9	5.4	Burke et al. (2002)
**Achene width**	3	ORS124, ORS488	49.3	43.3–65.2	10.2	Burke et al. (2002)
	6	ORS131	52.6	52.2–64.6	7.4	Burke et al. (2002)
	8	ORS894	59.1	50.7–75.0	9.2	Burke et al. (2002)
	9	ORS176	51.5	45.5–54.3	17.8	Burke et al. (2002)
	13	ORS388	0.0	0.0–6.0	11.0	Burke et al. (2002)
**Achene length**	2	ORS925	0.0	0.0–7.0	13.0	Baack et al. (2008)
	4	HT221	65.4	56.0–70.2	17.0	Baack et al. (2008)
	5	ORS852, ORS1120	45.3	37.3–52.3	16.9	Burke et al. (2002)
	5	ORS547	6.7	3.7–12.8	15.0	Baack et al. (2008)
	10	ORS684	41.3	37.3–48.7	10.7	Burke et al. (2002)
	13	HT568	0.0	0.0–24.3	15.0	Baack et al. (2008)
**Shattering**	4	HT298	0.0	0.0–4.0	10.7	Wills and Burke (2007)
	4	ORS674	33.4	32.6–41.4	6.4	Wills and Burke (2007)
	9	HT978	52.0	35.8–52.0	9.0	Baack et al. (2008)
	10	ORS437	15.8	7.8–20.1	9.0	Wills and Burke (2007)
	11	ORS5	31.0	23.0–45.7	6.6	Burke et al. (2002)
	13	HT568	0.0	0.0–6.7	13.0	Baack et al. (2008)
	16	ORS656	37.3	29.2–46.2	23.0	Baack et al. (2008)
	17	ORS811	41.2	29.4–44.6	5.0	Burke et al. (2002)
**Seed germination**	12	HT466	72.3	71.4–72.8	17.3	Wills and Burke (2007)
	15	ORS687	57.1	48.5–58.2	17.8	Wills and Burke (2007)
**% oil content**	4	ORS146	36.6	29.1–39.3	14.5	Burke et al. (2005)
**% palmitic acid**	6	HT769	26.3	18.4–41.7	15.8	Burke et al. (2005)
	17	ORS727	39.6	33.8–43.1	10.7	Burke et al. (2005)
**% stearic acid**	6	ORS374	24.3	20.3–28.3	35.9	Burke et al. (2005)
	10	ORS818	50.7	44.7–62.2	11.5	Burke et al. (2005)
**% oleic acid**	1	ORS718A, ORS474	19.5	3.3–25.5	10.1	Burke et al. (2005)
	3	ORS488	63.3	41.9–69.3	12.7	Burke et al. (2005)
	6	ORS374	22.3	16.4–28.3	24.0	Burke et al. (2005)
**% linoleic acid**	3	ORS124, ORS488	47.9	39.9–61.2	15.1	Burke et al. (2005)
	6	ORS374	24.3	18.4–28.3	28.9	Burke et al. (2005)
**Self-incompatibility**	17	ORS735	89.1	-	66.2	Gandhi et al. (2005)
**Self-pollination**	6	ORS349	75.4	-	2.6	Gandhi et al. (2005)
	15	ORS292	46.1	-	3.8	Gandhi et al. (2005)
	17	ORS735	88.1	-	57.5	Gandhi et al. (2005)
**Seed dormancy**	3	ORS1222	25.1	-	9.7	Gandhi et al. (2005)
	11	ZVG51	20.2	-	16.5	Gandhi et al. (2005)
	15	HT284	63.0	-	12.1	Gandhi et al. (2005)

^a^ 1-Logarithm of the odds (LOD) interval is shown, except for LOD interval reported by Dechaine et al. (2009) who presented 2-LOD interval; ^b^ Percentage of phenotypic variation explained (PVE) by a QTL.
